# Correlates of neurocognitive performance in older adults with chronic pain and negative emotions: baseline data from the problem adaptation therapy for pain (PATH-pain) randomized controlled trial

**DOI:** 10.3389/fpain.2024.1498283

**Published:** 2024-12-16

**Authors:** Irina Mindlis, Lisa D. Ravdin, M. Carrington Reid, Dimitris Kiosses

**Affiliations:** ^1^ Division of Geriatrics and Palliative Medicine, Weill Cornell Medicine, New York, NY, United States; ^2^ Department of Neurology, Weill Cornell Medicine, New York, NY, United States; ^3^ Department of Psychiatry, Weill-Cornell Institute of Geriatric Psychiatry, Weill Cornell Medicine, White Plains, NY, United States

**Keywords:** cognition, chronic pain, depressive symptoms, negative emotions, mild cognitive impairment, older adults

## Abstract

Chronic pain is highly prevalent among older adults, is associated with cognitive deficits, and is commonly treated in primary care. We sought to document the extent of impairment across specific neurocognitive domains and its correlates among older adults with chronic pain in primary care. We analyzed baseline data from the Problem Adaptation Therapy for Pain trial, which examined a psychosocial intervention to improve emotion regulation in 100 adults ≥ 60 years with comorbid chronic pain and negative emotions, who did not have evidence of moderate-to-severe cognitive impairment. Questionnaires on comorbidities, depressive symptoms, pain intensity, and pain-related disability were administered along with the Repeatable Battery for the Assessment of Neuropsychological Status (RBANS) and the Montreal Cognitive Assessment (MoCA). Multiple regression assessed the relationship between demographic and clinical characteristics with specific neurocognitive domains. Over half of participants (56%) had mild-to-moderate cognitive impairment (<26 on the MoCA). Across domains, participants scored the lowest in visuospatial/constructional (*M* = 86.2; *SD* = 15.7), and 15%–23% scored at least one standard deviation below the mean for immediate and delayed memory, visuospatial/constructional, and attention. In adjusted models, greater medical comorbidities were associated with poorer performance on the total RBANS, immediate memory, and attention. Cognitive deficits in older adults with chronic pain in primary care are substantial, with varying levels of deficits by neurocognitive domain. Future research should examine synergistic effects of chronic pain and comorbidities on cognition, and the impact of cognitive deficits on older adults' ability to engage in pain interventions and self-management behaviors.

## Introduction

In primary care, chronic pain is discussed in about 50% of visits with older adults, yet time for discussion is limited (1) as multiple priorities must get discussed during short visits. Among older adults, chronic pain and cognitive impairment are highly comorbid (2), with studies suggesting that older adults with chronic pain are twice as likely to report early cognitive decline, compared to those without chronic pain (3), and are at a higher risk for dementia (4, 5). Cognitive deficits may impact older adults' ability to engage in self-management behaviors necessary to manage chronic pain (6–8). However, the extent of this cognitive impairment and its correlates among older adults with chronic pain seen in primary care is understudied.

The relationship between chronic pain and cognitive impairment is well established across a wide range of cognitive tests (9, 10). Prior studies on community-dwelling older adults with chronic pain show that those with more severe pain or more pain-related interference perform worse on several cognitive domains, including memory, attention, and executive function compared to their peers with less or no pain (11, 12). For example, in a study on specific cognitive domains assessed by the Montreal Cognitive Assessment (MoCA) such as attention and orientation, older adults with chronic pain had lower MoCA scores compared to those without chronic pain in the domains of executive function, attention, memory, and language (13). The attentional hypothesis proposes that chronic pain impacts cognition by using up individual's resources. In short, pain may use up patients' cognitive resources by competing for attention, creating pain-related neuroplastic changes—for example, through reductions in thalamic and cortical gray matter associated with decreased pain inhibition (14), or leading to depression (15). As a result of these, patients' ability to conduct cognitive tasks such as sustaining attention or processing information is decreased (15, 16).

Given the high prevalence of chronic pain among older adults (17) and the potential implications of cognitive impairment on their ability to engage in pain self-management behaviors, we aimed to (1) characterize the degree of cognitive impairment in a sample of older adults with chronic pain receiving primary care, (2) ascertain deficits in specific cognitive domains, and (3) assess which factors are independently associated with deficits in specific cognitive domains.

## Methods

### Participants

Data for these analyses were collected during the baseline interview of the Problem Adaptation Therapy for Pain (PATH-Pain; NCT03487822) study, a randomized controlled trial testing the efficacy of a psychosocial intervention designed to improve emotion regulation to reduce pain-related disability, pain intensity, and depression in older adults ages ≥ 60 years with chronic pain and negative emotions. Full details of the study have been described elsewhere (18). Briefly, participants (*N* = 100) were recruited through an ambulatory geriatric primary care practice at an academic medical center in New York City through direct physician referrals, in-clinic flyers, and direct recruitment by research assistants in the waiting room between October 2017 and June 2019. In order to meet study eligibility, participants had to (1) report pain that was not due to cancer on most days for at least 3 months; (2) score ≥16 on the Montreal Cognitive Assessment (MoCA) (19); and (3) endorse negative emotions based on thresholds of the Positive and Negative Affect Schedule (PANAS-X: general negative affect subscore ≥ 20, fear subscore ≥ 10, hostility subscore ≥ 10, guilt subscore ≥ 10, or a sadness subscore ≥ 8) (20). Patients with moderate-to-severe cognitive impairment (i.e., MoCA score <16) or those with a recorded diagnosis of moderate-to-severe dementia in the medical record; and those who did not have sufficient English-language skills to participate were deemed ineligible. Baseline interviews were administered by trained research assistants following informed consent and included questionnaires and a neuropsychological battery (see Measures) administered in-person by the research assistants. Following this interview, patients were randomized into PATH-Pain (a collaborative program which includes a psychosocial intervention designed to improve emotion regulation) or usual care. The study protocol was approved by the institutional review board of Weill Cornell Medicine.

### Measures

#### Neurocognitive domains

The Repeatable Battery for the Assessment of Neuropsychological Status (RBANS) was used to assess neurocognitive functioning (21). The RBANS is a well validated brief cognitive battery (usually 20–30 min to administer) that measures functioning across five domains: Immediate Memory (ability to recall information immediately after it is presented), Visuospatial/Constructional (figure copy and line orientation), Language (ability to name line drawings and generate words to a semantic category), Attention (information processing and visual scanning), and Delayed Memory (anterograde memory capacity). In addition, a total score is computed by combining the five domain scores. RBANS indices on the 5 domains as well as Total RBANS score are based on a normal distribution scale (mean of 100, *SD* = 15), with higher values indicating better performance. Participants were also administered the MoCA at baseline, a brief measure which assessed global cognitive function over 8 areas of cognition (attention and concentration, executive functions, memory, language, visuoconstructional skills, conceptual thinking, calculations, and orientation). Scores below 26 indicate deficits in cognitive function. The MoCA is a widely used and well-validated measure with high sensitivity and specificity for detecting mild cognitive impairment and Alzheimer's disease (19).

#### Medical comorbidities

The Charlson Comorbidity Index was calculated through a review of each participant's electronic health record (22). A score of 0 means no comorbidities are present, higher scores indicate more severe comorbid conditions and a greater overall predicted mortality. In addition to the Charlson Comorbidity Index, the electronic health record was reviewed to abstract data regarding any neurocognitive condition, including mild cognitive impairment, memory impairment or disorder, dementia, Parkinson's disease, stroke, transient ischemic attack, head trauma, epilepsy or seizure disorder.

#### Depressive symptoms and negative emotions

The presence and severity of depressive symptoms were assessed through the Montgomery-Åsberg rating scale (MADRS), a 10-item scale widely used in studies of depressed older adults with and without cognitive impairment (23, 24). Overall scores range from 0 to 60, with higher scores indicating greater symptom severity. The PANAS-X (25) was used for the purposes of screening for eligibility. This scale consists of 60-items measuring the constructs of positive and negative affective states over the past week. Items consist of a number of words that describe different feelings and emotions (e.g., “cheerful” and “sad”), and respondents are asked to indicate to which extent they have felt that way over the past week, with answer choices in a Likert scale ranging from “very slightly or not at all” to “extremely”. Scores are calculated for each emotional subscale, and participants meeting validated cut off scores on at least one of the “negative emotions” scales (i.e., general negative affect, fear, hostility, guilt, or sadness) were eligible to participate.

#### Pain intensity

An 11-item numeric pain intensity scale was used gauge participants' average pain intensity level at the time of the baseline interview (0 = no pain, 10 = worst possible pain) (26).

#### Pain-related disability

A modified version of the Roland-Morris Disability Questionnaire was used to measure general pain-related disability (the original scale referred to back pain only) (27). This questionnaire asks individuals about the extent to which pain impacts daily functioning on the day it is administered. Scores can range from 0 (none) to 24 (severe), with higher scores indicating greater pain-related disability.

#### Pain conditions and treatment

Number of pain conditions and use of opioid medications were obtained both via self-report during the baseline interview, as well as through chart reviews of the electronic health record by trained RAs for completion. Discrepancies were resolved by relying on patients' self-reports.

#### Sociodemographic measures

Sociodemographic variables collected included age, gender, race and ethnicity, marital status, and educational level.

#### Data analysis

Following descriptive statistics, univariate association of clinical and demographic characteristics (education, comorbidities, depressive symptoms, pain intensity, number of pain conditions, opioid use, pain-related disability) with specific neurocognitive domains (immediate memory, visuospatial, language, attention, delayed memory) were assessed using linear regression. Variables significantly associated with specific neurocognitive domains in univariate analyses were then examined through multiple regression analysis, testing the relationship between these clinical and demographic characteristics with specific neurocognitive domains. All analyses were performed using two-tailed tests with significance set at *p* < .05 level in SPSS version 29 (28). A sample of 98 would have been needed to allow for power to detect an adjusted, standardized regression coefficient as low as .15 as calculated through G*Power 3.1 (29).

## Results

Participants (*N* = 100) were largely female (80%), with an average age of 75.5 (*SD* = 8.9). The sample was diverse in terms of race/ethnicity: 67% identified as White, 15% as Black or African American, 8% as Multiracial, 3% as Asian, and 3% as Other, while 8% of participants identified as Hispanic or Latine. The sample had a mean educational level of 15.9 years (*SD* = 2.7). About a quarter of participants were married or partnered (24%), and another quarter were divorced or separated (27%), with the remaining participants reporting being widowed (19%) or never married (20%). Based on the MADRS, 76% of patients evidenced mild depressive symptoms, 12% had moderate depression, and another 12% had no depression. For full participant characteristics, see Table 1.

**Table 1:** Sociodemographic and clinical characteristics of study participants.

Variable	All participants (*N* = 100)	MoCA ≥ 26 (*N* = 44)	MoCA < 26 (*N* = 56)	*p*-value
Female,%	80 (80)	36 (82%)	44 (79%)	.687
Age, *M* (*SD*)	75.5 (8.9)	74.7 (7.1)	76.1 (10.2)	.396
Race, *N* (%)				.178
Black or African American	15 (15)	4 (9%)	11 (20%)	
Asian	3 (3)	1 (2%)	2 (4%)	
Multiracial	8 (8)	1 (2%)	7 (12%)	
Other	3 (3)	1 (2%)	1 (2%)	
White	67 (67)	35 (79%)	32 (57%)	
Hispanic/Latine, *N* (%)	8 (8)	4 (9%)	5 (9%)	.978
Marital status, *N* (%)				.903
Married/partnered	24 (24)	10 (23%)	14 (25%)	
Widowed	19 (19)	9 (20%)	10 (18%)	
Divorced/separated	27 (27)	11 (25%)	16 (29%)	
Never married	30 (20)	14 (32%)	16 (29%)	
Years of education, *M* (*SD*)	15.9 (2.7)	16.9 (2.2)	15.2 (2.8)	.001**
Charlson Comorbidity Index, *M* (*SD*)	3.6 (3.1)	3.0 (3.4)	4.1 (2.7)	.084
Pain intensity, *M* (*SD*)	4.7 (2.6)	4.7 (2.5)	4.7 (2.7)	.966
Pain-related disability, *M* (*SD*)	13.4 (4.9)	11.9 (4.8)	14.5 (4.8)	.011*
Number of pain conditions, *M (SD)*	2.6 (1.4)	2.6 (1.1)	2.6 (1.5)	.857
Opioid medications, *N (%)*	23 (23)	8 (19%)	15 (30%)	.229
Depressive symptoms, *M* (*SD*)	12.2 (5.5)	12.0 (5.3)	12.3 (5.7)	.771
RBANS total, *M* (*SD*)	92.9 (13.7)	101.8 (12.0)	85.9 (10.7)	<.001**
Immediate Memory, *M* (*SD*)	96.0 (15.4)	104.7 (11.8)	89.2 (14.6)	<.001**
Visuospatial/Constructional, *M* (*SD*)	86.2 (15.7)	93.6 (12.2)	80.4 (15.9)	<.001**
Language, *M* (*SD*)	101.0 (13.1)	104.9 (13.9)	98.0 (11.7)	.008**
Attention, *M* (*SD*)	100.1 (15.5)	103.8 (17.1)	97.1 (13.6)	.030*
Delayed Memory, *M* (*SD*)	91.3 (16.9)	101.0 (11.1)	83.7 (16.9)	<.001**

Four participants refused to provide racial identity information; one participant refused ethnicity. *T*-tests compared participants below the cut off for the MoCA with participants above the cut-off for the MoCA.

*p < .05.

**p < .01.

Based on total MoCA scores (19), 56% scored below the threshold for normal cognitive function (less than 26), indicating mild-to-moderate cognitive impairment. Table 1 shows that participants scoring below (vs. above) this threshold were more likely to have fewer years of education (*t* = 3.3, *p* < .001) and greater pain-related disability (*t* = −2.59, *p* = .011). Of note, even when excluding participants with a chart-documented history of a neurocognitive condition (i.e., mild cognitive impairment, memory impairment or disorder, dementia, Parkinson's disease, stroke, transient ischemic attack, head trauma, epilepsy or seizure disorder; *n* = 27) and those suspected of poor effort on the RBANS (30) (*n* = 1), 52% still scored below the cut-off for the MoCA, suggesting mild cognitive impairment.

In terms of specific neurocognitive domains assessed on the RBANS, mean scores ranged from 86.2 (*SD* = 15.7) for visuospatial/constructional abilities, with the highest mean scores in the language domain (mean = 101.0, *SD* = 13.1). We additionally examined the proportion of participants who scored below one and two standard deviations from the norm on the RBANS (standard scores have a mean of 100 and a standard deviation of 15). Full results appear in Table 2. Except for the language domain, 15%–23% of participants were one standard deviation below the mean for immediate and delayed memory, visuospatial/constructional, and attention—which is notable especially considering the high educational levels of this sample. Further, in the domains of delayed memory and visuospatial/constructional, 16% and 21% of participants scored at least two *SD* below the norm, respectively (suggesting moderate impairment).

**Table 2:** Performance in neurocognitive domains (*N* = 100).

Variable	All Participants *M* (*SD*)	Within the mean (RBANS score > 85)%	1 *SD* below mean (RBANS score > 70 and < 85)%	2 *SD* below mean (RBANS score < 70)^a^%
RBANS Total	92.9 (13.7)	72%	21%	7%
RBANS Immediate Memory	96.0 (15.4)	76%	17%	7%
RBANS Visuospatial/Construction	86.2 (15.7)	56%	23%	21%
RBANS Language	101.0 (13.1)	95%	3%	2%
RBANS Attention	100.1 (15.5)	77%	22%	1%
RBANS Delayed Memory	91.3 (16.9)	69%	15%	16%

^a^ Scores < 70 suggest moderate impairment in the RBANS domain.

In univariate analysis (Table 3), performance on the total RBANS was higher in those with higher levels of education (*β* = .49, *p* < .001) and fewer comorbidities (*β* = −.36, *p* < .001). Similarly. better performance on the immediate memory domain was associated with greater education (*β* = .40, *p* < .001), and having fewer comorbidities (*β* = −.29, *p* = .005). In the case of the visuospatial/constructional domain, performance was greater in those with higher levels of education (*β* = .408, *p* < .001), greater depressive symptoms (*β* = .31, *p* = .002), and a greater number of pain conditions (*β* = .24, *p* = .020). On the language index, performance was better among those with higher levels of education (*β* = .32, *p* < .001), fewer comorbidities (*β* = .23, *p* = .031), and less pain-related disability (*β* = .29, *p* = .004). In the case of the attention index, better performance was associated with higher education (*β* = .27, *p* = .006) and fewer comorbidities (*β* = −.28, *p* = .008). Finally, performance on the delayed memory domain was associated only with education levels (*β* = .31, *p* < .001). Notably, we did not find significant associations between neurocognitive domain performance with pain intensity or opioid use.

**Table 3:** Univariate associations with neurocognitive domains (*N* = 100).

Variable	RBANS Total		Immediate memory		Visuospatial		Language		Attention		Delayed memory	
	Estimate	*p*	Estimate	*p*	Estimate	*p*	Estimate	*p*	Estimate	*P*	Estimate	*p*
Education	.493	<.001**	.398	<.001**	.408	<.001**	.317	.001**	.274	.006**	.314	.001**
Comorbidities	−.356	<.001**	−.295	.005**	−.206	.051	−.227	.031*	−.280	.008**	−.188	.076
Depressive symptoms	.096	.343	−.024	.813	.312	.002**	.081	.424	−.038	.706	−.018	.858
Pain intensity	.040	.695	.128	.206	.022	.829	−.153	.128	.016	.876	.113	.265
Number of pain conditions	.144	.176	.049	.647	.245	.020*	−.041	.705	.097	.365	.119	.264
Opioid use	−.133	.213	−.105	.324	−.012	.914	−.202	.056	−.039	.718	−.106	.319
Pain-related disability	−.166	.100	−.067	.510	−.038	.704	−.289	.004**	−.106	.293	−.085	.401

* *p* < .05.

** *p* < .01. Standardized coefficients.

Subsequently, multiple regression models were constructed entering all covariates significantly associated with specific neurocognitive domains in univariate analysis (education, comorbidities, depressive symptoms, number of pain conditions, and pain-related disability). Separate regression models were constructed for the total RBANS score, as well as for each neurocognitive domain (immediate memory, visuospatial/constructional, language, attention, and delayed memory). Detailed results for each model are shown in Table 4.

**Table 4:** Multiple regression evaluating adjusted associations with neurocognitive domains (*N* = 100).

Variable	RBANS total		Immediate memory		Visuospatial		Language		Attention		Delayed memory	
	*β*	*p*	*β*	*p*	*β*	*p*	*β*	*p*	*β*	*p*	*β*	*p*
Education	.414	<.001**	.354	<.001**	.353	<.001**	.266	.011*	.209	.054	.273	.013*
Comorbidities	−.234	.015*	−.217	.037*	−.101	.295	−.125	.223	−.219	.044*	−.110	.307
Depressive symptoms	.116	.214	−.025	.805	.312	.001**	.148	.143	−.032	.759	−.016	.879
Number of pain conditions	.052	.562	−.027	.786	.156	.099	−.092	.356	.048	.645	.068	.517
Pain-related disability	−.092	.333	.031	.765	−.048	.617	−.256	.015*	−.032	.767	−.026	.810
*R²*	.321		.202		.302		.202		.128		.117	

* *p* < .05.

** *p* < .01. Standardized coefficients.

### Total RBANS

In the case of the total RBANS score, higher levels of education (*β* = .41, *p* < .001) and fewer comorbidities (*β* = −.23, *p* = .015) remained independently associated with higher total RBANS scores. Total RBANS score was not associated with depressive symptoms, number of pain conditions, or pain-related disabilities.

### Immediate memory

Better performance on the immediate memory domain remained associated with greater education (*β* = .35, *p* < .001) and fewer comorbidities (*β* = −.22, *p* = .037). We did not find any significant relationships between immediate memory and depressive symptoms, number of pain conditions, or pain-related disability.

### Visuospatial/constructional

The visuospatial/constructional domain remained associated with greater education (*β* = .35, *p* < .001) as in univariate analysis and, surprisingly, it remained associated with greater depressive symptoms (*β* = .31, *p* < .001)—contrary to our expectations. The association between number of pain conditions and visuospatial/constructional abilities was no longer significant, and there was also no association with comorbidities or pain-related disability.

### Language

Better performance on the language domain remained associated with education (*β* = .27, *p* = .011) and lower pain-related disability (*β* = −.26, *p* = .015), yet comorbidities were no longer significantly associated with language in adjusted analysis. There was also no significant association with number of pain conditions or depressive symptoms.

### Attention

Better performance on the attention domain was associated only with fewer comorbidities (*β* = −.22, *p* = .044), as the association with higher education was not significant in the adjusted model.

### Delayed memory

Finally, greater performance on the delayed memory domain remained associated solely with education levels (*β* = .27, *p* = .013) as in univariate analysis.

## Discussion

In this sample of older adults with chronic pain and negative emotions recruited from an academic primary care practice, we found that over half of all participants evidenced mild-to-moderate cognitive impairment; and over a quarter showed reduced scores on visuospatial/constructional abilities and delayed memory (followed by immediate memory and attention). In adjusted models, we additionally found comorbidities were associated with reduced cognitive performance, and specifically with lower performance on the immediate memory and attention domains. These findings are noteworthy as patients with no chart-recorded diagnoses of neurocognitive impairment may nevertheless have neurocognitive deficits that could impair overall functioning and limit their ability to engage in pain self-management behaviors.

Cognitive impairment has been reported to occur more frequently in older adults with a greater number of chronic illnesses (31, 32). For example, among participants with two or more chronic illnesses or *multimorbidity*, the odds of mild cognitive impairment or cognitive impairment with no dementia are twice as high compared to those without multimorbidity (33). Possible explanations for this relationship include the greater prevalence of polypharmacy and disease-disease interactions common in multimorbidity, which have been associated with cognitive impairment (33). While research into multimorbidity, chronic pain, and cognitive impairment has been more limited, emerging evidence suggests higher global cognitive functioning is associated with lower odds of pain that limits daily activities among older adults with multimorbidity (34). Similarly, the risk for incident Alzheimer's disease and related dementias is higher in those with chronic pain and a greater number of comorbidities (35). Future work is needed to understand the independent and potentially synergistic effects of multimorbidity and chronic pain on the development of cognitive impairment and poor performance on specific neurocognitive domains.

Unsurprisingly, we found that greater educational attainment was associated with better performance on almost all neurocognitive domains in adjusted analysis, in line with large epidemiological samples of older adults (36) and meta-analyses (37). While likely associated with cognitive function in older adults through multiple pathways, prior research suggests that education contributes to individual differences in cognitive skills that emerge in early adulthood, but persist into old age (38). In contrast, we found that higher depressive symptoms were associated with better performance in the visuospatial/constructional domain. Several explanations may account for this finding. Scoring for the visuospatial/constructional domain of the RBANS depends on two of the battery's subtests: Figure Copy, and Line Orientation (21). Prior studies have shown that older adults may perform worse in the visuospatial/constructional domain compared to the other RBANS domains due to an overly strict and subjective scoring procedure (39, 40). Specifically, there is a concern that the Figure Copy subtest penalizes even healthy older adults with overly harsh scoring of even minor inaccuracies in the copying process. While scores are based on age-adjusted population norms, Figure Copy subtest scores vary considerably: at the mean age of our sample (75 years), the sample's mean Figure Copy score of 15 corresponds to the 6th percentile of performance. However, a score of 16 would have been considered “within normal limits”, at the 16th percentile. This great variability at small score ranges may have resulted in lower scores than expected on this specific domain for our sample. Other potential explanations for this association is that most participants in our sample had mild depressive symptoms, with few scoring in the “no depression” or “moderate” range of the MADRS (23). Therefore, it is possible that the limited variability in depression scores impacted our findings—despite the use of negative emotions as an inclusion criterion into the study. Further research is needed to examine the association between depressive symptoms and visuospatial/constructional skills in other samples of older adults with chronic pain, including the use of the modified RBANS scoring criteria to adjust for overly strict scoring (39).

We did not find an association between opioid medication use and cognitive function. Incidence of dementia has been found to be higher in chronic pain patients using opioid medications, compared to those not on opioids (41). Similarly, among older adults, long-term opioid medication use was associated with poorer performance on global measures of cognition—yet was not predictive of performance on tasks assessing immediate and delayed memory, working memory, verbal ability, psychomotor speed, or executive function (42). A recent review highlights the mixed findings for the association between opioid use and cognition, citing that most studies showed no effect of opioid use, and a smaller number showed mixed effects (43). These mixed findings highlight the importance of further work examining opioid use on specific neurocognitive domains. It is also possible that, unlike some prior work showing detrimental effects of opioid use on attention, language, orientation, and memory in those with higher opioid exposures (43), we were limited by our use of a binary measure of opioid medication use that solely indicated the use of a prescription medication (through both self-report and medication chart reviews). As such, we were not able to look at a potential dose-response relationship between higher opioid use and cognitive impairment. Similarly, we did not assess the time since initiation of opioid medications, nor record specific opioid medications.

A proposed mechanism for the relationship between pain and performance on cognitive function tests is the attentional hypothesis, by which pain competes for attentional resources in the brain and leaves those with persistent pain “depleted” (15, 16). We did not find a significant association between pain intensity and cognitive function, and only a small association between pain-related disability and language. Prior cross-sectional studies of community-dwelling older adults with chronic pain have similarly reported no association between pain intensity and global cognition or specific neurocognitive domains once models are adjusted for demographic, clinical characteristics, and attention (11). Further, they report only modest associations between pain-related disability and memory, but not executive function or attention. These prior findings have been suggested to provide support for the attentional hypothesis, where the distraction created by the study's cognitive tasks attenuates the experience of pain. It is also possible that the attentional demands from pain have a cumulative effect over time, and are not evident in cross-sectional studies. For example, in a large cohort study of older adults with persistent pain across study waves, pain intensity was associated with a faster rate of cognitive decline in a dose-response manner for global cognition, verbal memory, semantic fluency, and temporal orientation (44). Future work is needed to further disentangle these relationships, as well as identify who may be better able to gain from the use of distraction techniques to enhance pain management, vs. interventions aimed at reducing the interference of pain with valued activities. Additionally, future work should examine other frameworks such as the fatigue hypothesis by which chronic pain impacts motivational rewards pathways in the brain and how this might explain our findings (45).

Our findings must be interpreted in the context of several limitations. First, we acknowledge that educational levels were high in our sample, and likely the results would have been different in a sample with lower levels of educational attainment or greater variability—at the same time, our findings of reduced scores across multiple cognitive domains is especially noteworthy for a sample with such high levels of educational attainment. The sample being overwhelmingly female also limits our generalizability and limited our ability to examine these findings by sex. Similarly, while the presence of negative emotions was an inclusion criterion, most participants had mild depressive symptoms, with few scoring in the “no depression” or “moderate” range of the MADRS (23). Both the selection by negative emotions and the limited variability of depressive symptoms may have impacted our findings regarding depressive symptoms and cognitive function, including the surprising finding that those with higher depressive symptoms performed better in visuospatial/constructional tasks. Relatedly, the selection by negative emotions limits our ability to generalize to samples of older adults with chronic pain with lower levels of negative emotions. Another factor that impacts our findings is the limited sample size. While we were powered to detect moderate effects, it is possible that a larger sample would have been able to explore additional covariates and lead to more robust estimates of associations. Relatedly, our cross-sectional design limits us from making inferences regarding temporality—however, evidence suggests that the relationships between chronic pain and cognitive function are bidirectional (10, 46). As this was a secondary analysis of a randomized controlled trial for older adults with chronic pain and negative emotions, we did not have a healthy control group. Finally, while we used a well-validated and widely used measure of comorbidities, the Charlson Comorbidity Index was developed to measure mortality risk, and thus may not be the best approach to capturing comorbidities relevant to neurocognitive functioning. Nevertheless, this study had several strengths, including the focus on a medically complex sample of older adults with comorbid chronic pain and negative emotions, and the use of the RBANS administered in-person by trained research assistants instead of relying solely on a global screening measure of cognitive function.

Our findings have several implications for clinical encounters with older adults with chronic pain. Despite the exclusion of participants with MoCA scores indicating moderate-to-severe cognitive impairment (16 or less), and even when excluding those with neurocognitive conditions on their medical records, most participants in the sample evidenced at least mild cognitive impairment (based on MoCA cut-off scores). Thus, while these patients do not have a recorded neurocognitive disorder on their charts, our results show they have neurocognitive deficits that may impair functioning. While neuropsychological assessments are not part of usual care in outpatient primary care and pain management settings, cognitive decline can contribute to undertreatment and disability, and poor quality of life among older adults with chronic pain (47). Referring affected individuals for neurocognitive testing could help to shed light on deficits in specific neurocognitive domains beyond what can be ascertained through the use of global cognitive screening measures that are typically employed in primary care settings. Certain neurocognitive domains we assessed (e.g., attention, immediate and delayed memory) have special relevance to medical encounters and may affect ability to engage in the clinical encounter, and to recall information following the encounter. Further work is needed to understand whether these deficits contribute to treatment non-adherence among patients with chronic pain through the potentially mediating role of difficulty engaging during medical appointments and deficits in information recall. Finally, similar considerations should apply to engagement with multi-component, longitudinal interventions common for pain management, such as cognitive behavioral therapy for pain, mindfulness interventions, and acceptance and commitment therapy. Future work is needed to understand whether these interventions may prove more difficult for older adults with chronic pain and cognitive impairment, and whether deficits in specific neurocognitive domains affects their ability to master the skills taught by behavioral interventions for pain.

## Data Availability

The data analyzed in this study is subject to the following licenses/restrictions: Consent was not sought from patients to share datasets resulting from this study. Requests to access these datasets should be directed to irm4004@med.cornell.edu.
